# Analyzing rating distributions with heaps and censoring points using the generalized Craggit model

**DOI:** 10.1016/j.mex.2020.100868

**Published:** 2020-03-19

**Authors:** Volker Lang, Martin Groß

**Affiliations:** a Faculty of Sociology, Bielefeld University, Bielefeld, Germany; b Institute of Sociology, Tübingen University, Tübingen, Germany

**Keywords:** Censored data, Heaped data, Craggit model, Structural equation model, Rating instruments, Response scales, Sequential responses, Survey experiments

## Abstract

In this article, we introduce a new, highly flexible model to analyze distributions with heaps and censoring points, which we call the generalized Craggit model. Distributions with heaps and censoring points can be found in many social science applications. For example, such distributions can be the result of sequential or multistep rating processes. Our model is a combination of a Craggit model and a generalized ordered probit model. It can account for multiple heaps and censoring points in distributions. We used this model to analyze a factorial survey experiment on earnings justice attitudes in the SOEP-Pretest 2008. In this experiment, a three-step rating instrument was used, which resulted in a rating distribution with heaps and censoring. Our generalized Craggit model fits the data of this experiment much better than a hierarchical linear model, which is the method that is usually implemented to analyze factorial survey experiments.

## Highlights

• The generalized Craggit model allows multiple heaps and censoring points to be accounted for in distributions.
• The generalized Craggit model can be used to adequately analyze sequential or multistep rating processes.
• In an example application, the generalized Craggit model fits the data analyzed much better than a standard hierarchical linear model.

## Graphical abstract

**Figure:**
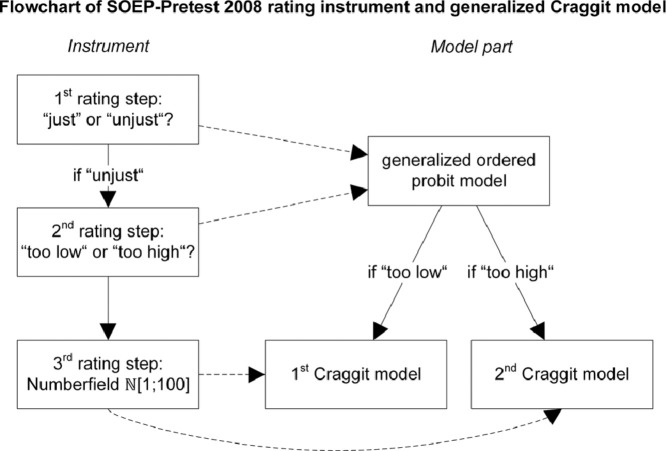

**Table:** Specifications Table

Subject Area:	Social Sciences
More specific subject area:	Factorial Survey Experiments
Method name:	Generalized Craggit Model
Name and reference of original method:	Cragg, J.G. (1971). Some statistical models for limited dependent variables with application to the demand for durable goods. Econometrica 39(5):829–844.
Resource availability:	The SOEP-Prestest 2008 is available as a scietific use file here: https://www.diw.de/en/diw_02.c.222517.en/data.html
	All analyses in the publication have been conducted with the statistical software Stata (version 14).

## Method Details

We conducted a study on the influences of gender-specific status beliefs on earnings justice attitudes [11]. For our analyses, we used a factorial survey experiment on earnings justice attitudes included in the SOEP-Pretest 2008 with 1066 respondents who overall rated 26,650 vignettes [16].^1^ For further details on the design and implementation of this factorial survey experiment, see [11], [16] and [15]. To adequately analyze the data of this factorial survey experiment, we developed a so-called generalized Craggit model, which we introduce in the remainder of this paper.

Figure A1 in the supplementary materials shows an example vignette of the factorial survey in the SOEP-Pretest 2008. It shows that the response instrument used implements a three-step rating process. First, respondents classify a vignette as either “just” or “unjust”. Second, if they classify it as “unjust”, they categorize it as “unjustly too high” or “unjustly too low”. Third, they are instructed to fill in a number between 1 and 100 expressing the degree of injustice. After a zero is assigned to vignettes rated as “just” and the signs are changed for ratings classifying vignettes as “unjustly too low”, all ratings can be expressed on a joint scale ranging from −100 to 100.

**Fig. 1:**
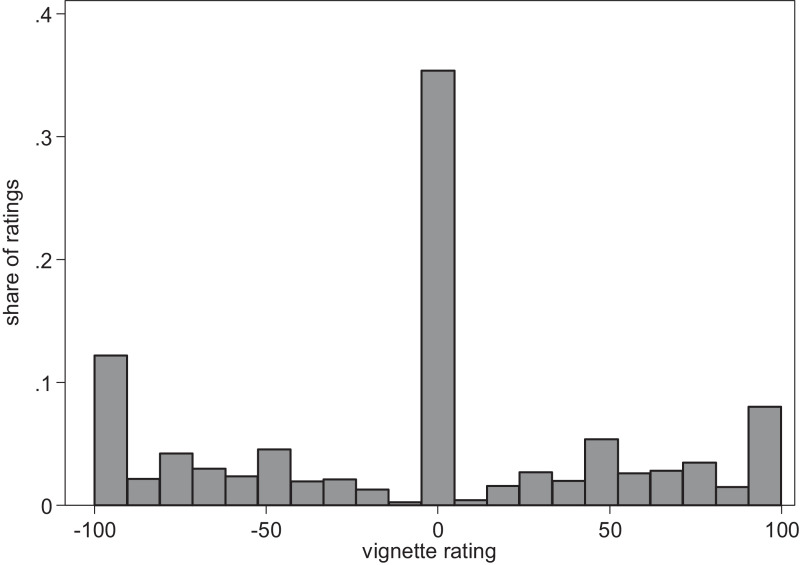
Distribution of vignette ratings in the SOEP-Pretest 2008

Fig. 1 displays the realized vignette rating distribution on this scale. The distribution shows three major heaps at 0 (vignettes rated as “just”; 34.8% of the ratings), −100 (vignettes rated as “unjustly much too low”; 11.7% of the ratings), and 100 (vignettes rated as “unjustly much too high”; 7.9% of the ratings), as well as several minor heaps (for example, at 50; 5.0% of the ratings). [15] conduct analyses that show that the less fine-grained vignette ratings causing these heaps are related neither to specific parts of the experiment (for example, the beginning or the end) nor to the age or education of respondents. They conclude that it is not necessary to implement stepwise or more fine-grained rating instruments in factorial survey experiments, since in most cases, attitudes are not expressed in such detailed ratings. However, the findings of [15] can also be interpreted to show that most respondents adapt the granularity of their ratings in a way that matches a stepwise rating process, starting coarsely (which causes the heaps) and adding more detail if possible and deemed necessary. The standard tool to analyze factorial surveys—a hierarchical linear regression model [5]—cannot be used to adequately analyze a multistep rating process, resulting in a rating distribution with heaps and censoring points similar to the one displayed in Fig. 1.^2^

Own calculations based on SOEP-Pretest 2008 (N_vignettes_ = 26,650; N_respondents_ = 1066).

## The generalized Craggit model

Instead, to account for the three-step rating process and the related heaps in the rating distribution, we combine the Craggit model [3] with a generalized ordered probit model [13,17], which we call the generalized Craggit model.^3^

Table 1 summarizes the following remarks on how we translate the three rating steps into a generalized Craggit model. In combination, the first and second rating steps classify the justice evaluations into three rather crude categories: earnings are rated as “unjustly too low” (< 0), “just” (0) or “unjustly too high” (> 0). To estimate the effects of explanatory variables on this classification, we use a generalized ordered probit model consisting of two probit equations:^4^

(1) $$y_{v}^{1}\geq \text{invprobit}\left(y_{v}^{*,1}=\alpha^{l}+{X_{v,i}}^{l}*\beta^{x,l}+\beta^{\text{ear},l}*\ln{\left(\text{earnings}\right)}_{v}+\varepsilon_{i}^{1}+\varepsilon_{v}^{l}\right)$$

(2) $$y_{v}^{h}\geq \text{invprobit}\left(y_{v}^{*,h}=\alpha^{h}+X_{v,i}^{h}*\beta^{x,h}+\beta^{\text{ear},h}*\ln{\left(\text{earnings}\right)}_{v}+\varepsilon_{i}^{h}+\varepsilon_{v}^{h}\right)$$

**Table 1:** Translation of rating steps in SOEP-Pretest 2008 into a generalized Craggit model.

*1st and 2nd rating step*: “unjustly too low” (< 0) vs. “just” (0) vs. “unjustly too high” (> 0) → *Generalized ordered probit model*:
Eq. (1) (Probit model): “unjustly too low” (< 0) vs. “just or unjustly too high” (≥ 0)
Eq. (2) (Probit model): “unjustly too low or just” (≤ 0) vs. “unjustly too high” (> 0)
*3rd rating step* given that rating after 2nd rating step was “unjustly too low” (< 0) → *1st Craggit model*:
Eq. (3) (Probit model): “unjustly much too low” (−100) vs. “unjustly too low” (ℕ[−99;−1])
Eq. (4) (Truncated regression model): Detailed ratings in range ℕ[−99;−1]
*3rd rating step* given that rating after 2nd rating step was “unjustly too high” (> 0) → *2nd Craggit model*:
Eq. (5) (Probit model): “unjustly much too high” (100) vs. “unjustly too high” (ℕ[1;99])
Eq. (6) (Truncated regression model): Detailed ratings in range ℕ[1;99]

Building on the two ordered indicator variables y_v_^l^ and y_v_^h^, Eq. (1) expresses the choice between “unjustly too low and just” or “unjustly too high” using a probit model (in which y_v_^l^ = 1 if y_v_ ≥ 0 and y_v_^l^ = 0 if y_v_ < 0), and Eq. (2) formulates the choice between “unjustly too low” or “just and unjustly too high” using another probit model (in which y_v_^h^ = 1 if y_v_ > 0 and y_v_^h^ = 0 if y_v_ ≤ 0). invprobit is an inverted probit-function, y_v_*^,l^ and y_v_*^,h^ are the estimated values of the observed outcomes on their respective latent probit scales, α^l^ and α^h^ are fixed overall intercepts, X_v,i_^l^ and X_v,i_^h^ are matrixes of explanatory variables on the vignette level v and the respondent level i, β^x,l^ and β^x,h^ are vectors of coefficients for the explanatory variables, ln(earnings)_v_ is the natural logarithm of the earnings stated in the vignettes,^5^ β^ear,l^ and β^ear,h^ are the coefficients associated with the log earnings, ε_i_^l^ and ε_i_^h^ are normally distributed random intercepts on the respondent level, and ε_v_^l^ and ε_v_^h^ are normally distributed error terms on the vignette level. This first ordered categorical part of the generalized Craggit model (Eqs. (1) and (2)) addresses the heap of “just”-rated (0) vignette scenarios. It facilitates adequate modeling of the latent variance between the “unjust too low”, “just” and “unjust too high” categories of ratings.

The third rating step involves the more fined-grained ratings. However, our model needs to address the heaps of “unjustly much too low” (−100) and “unjustly much too high” (100) ratings in the rating distribution. These heaps represent censoring points: people choose these extreme values if they think that the provided scale is not useful to express a gradation of injustice. These vignette scenarios are evaluated as extremely unjust. Consequently, a linear regression of explanatory variables on the gradation of unjustness is only sensible if these extreme evaluations are not included. To model this censored rating process, we implement two Craggit models. Each of these Craggit models consists of two equations. The first Craggit model captures the subsequent rating process for vignettes that are rated “unjustly too low” (y_v_^l^ = 0) after the first and second rating step:

(3) $$y_{v}^{\text{ml}}\geq \text{invprobit}\left(y_{v}^{*,\text{ml}}=\alpha^{\text{ml}}+X_{v,i}^{\text{ml}}*\beta^{x,\text{ml}}+\beta^{\text{ear},\text{ml}}*\ln{\left(\text{earnings}\right)}_{v}+\varepsilon_{i}^{\text{ml}}+{\varepsilon_{v}}^{\text{ml}}\right)$$

(4) $$y_{v}^{\text{tl}}=\text{truncreg}\_\text{below}\left(y_{v}^{*,\text{tl}}=\alpha^{\text{tl}}+X_{v,i}^{\text{tl}}*\beta^{x,\text{tl}}+\beta^{\text{ear},\text{tl}}*\ln{\left(\text{earnings}\right)}_{v}+\varepsilon_{i}^{\text{tl}}+\varepsilon_{v}^{\text{tl}}\right))$$

Here, y_v_^ml^ in Eq. (3) is an indicator variable related to the second step of the rating process to differentiate vignettes that are rated “much too low”, conditional on being rated “too low” (i.e., y_v_^ml^ = 1 if y_v_ > −100 and y_v_^l^ = 0, and y_v_^ml^ = 0 if y_v_ = −100), and y_v_^tl^ in Eq. (4) is a variable containing the ratings of vignettes that are rated “unjustly too low” but not “much too low” (i.e., y_v_^tl^ = ℕ[−99;−1]). truncreg_below is a truncated regression function for scales that are left truncated, and y_v_*^,tl^ are the estimated values of the observed outcomes on the respective latent truncated regression scale. The remainder of the notation in Eqs. (3) and (4) is interpreted analogously to the notation in Eqs. (1) and (2) (see above).

Similarly, the second Craggit model captures the subsequent rating process for vignettes that are rated “unjustly too high” (y_v_^h^ = 1) after the first and second rating step:

(5) $$y_{v}^{\text{mh}}\leq \text{invprobit}\left(y_{v}^{*,\text{mh}}=\alpha^{\text{mh}}+X_{v,i}^{\text{mh}}*\beta^{x,\text{mh}}+\beta^{\text{ear},\text{mh}}*\ln{\left(\text{earnings}\right)}_{v}+\varepsilon_{i}^{\text{mh}}+\varepsilon_{v}^{\text{mh}}\right)$$

(6) $$y_{v}^{\text{th}}=\text{truncreg}\_\text{above}\left(y_{v}^{*,\text{th}}=\alpha^{\text{th}}+X_{v,i}^{\text{th}}*\beta^{x,\text{th}}+\beta^{\text{ear},\text{th}}*\ln{\left(\text{earnings}\right)}_{v}+\varepsilon_{i}^{\text{th}}+\varepsilon_{v}^{\text{th}}\right))$$

Here, y_v_^mh^ in Eq. (5) differentiates vignettes that are rated “much too high”, conditional on being rated “too high” (i.e., y_v_^mh^ = 1 if y_v_ < 100 and y_v_^h^ = 1, and y_v_^mh^ = 0 if y_v_ = 100),^6^ and y_v_^th^ in Eq. (6) contains ratings of vignettes that are rated “unjustly too high” but not “much too high” (i.e., y_v_^th^ = ℕ[1;99]). truncreg_above is a truncated regression function for scales that are right truncated. The remainder of the notation in Eqs. (5) and (6) is interpreted analogously to the notation in Eqs. (3) and (4) (see above).

Each of the six equations in the generalized Craggit model has a respondent-level random intercept, and additionally, the model contains the covariances between these random-level intercepts.^7^ The variance-covariance matrix (COV) of these respondent-level random intercepts is given by the following equation:

(7) $$\text{COV}\left(\varepsilon_{i}^{l},\varepsilon_{i}^{h},\varepsilon_{i}^{\text{ml}},\varepsilon_{i}^{\text{mh}},\varepsilon_{i}^{\text{tl}},\varepsilon_{i}^{\text{th}}\right)$$

Eqs. (1) to (7) are jointly estimated using a maximum likelihood algorithm. The error terms in the probit Eqs. (1), (2), (3) and (5) are fixed to one to identify the respective parts of the model. Thus, our generalized Craggit model is a specific form of a generalized multilevel structural equation model (GSEM, [14]), building on earlier GSEMs used to analyze factorial surveys [10].

Like all GSEMs, the generalized Craggit model supports the specification of constraints between parameters. To implement constraints between parameters across equations, we have to set the additional restriction that the variances of the error terms of the truncated regression Eqs. (4) and (6) are equal (VAR(ε_v_^tl^) = VAR(ε_v_^th^)). With this restriction in place, the scales of the probit and the truncated regression equations can be mapped on each other based on the ratio of the standard deviations of their error terms [10,12]. Specifically, since the standard deviations of the error terms are one for the probit equations and the standard deviations of the error terms are equal for the two truncated regression equations, this ratio is given by 1 / sqrt(ε_v_^tl^) = sqrt(ε_v_^tl^). Therefore, coefficients can either be constrained across equations on the probit scale (β^x,truncreg^ / sqrt(ε_v_^tl^) = β^x,probit^) or on the truncated regression scale (β^x,truncreg^ = β^x,probit^ * sqrt(ε_v_^tl^)).

Furthermore, the generalized Craggit model is a GSEM implementation of the justice evaluation function developed by Jasso [6,^7^,9]:

(8) $$y_{v}=\alpha +X_{v,i}*\beta^{x}+\beta^{\text{ear}}*\ln{\left(\text{earnings}\right)}_{v}+\varepsilon_{i}+\varepsilon_{v}$$

Here, y_v_ are the justice ratings of the vignettes, and the rest of the notation in Eq. (8) is interpreted analogously to the notation in Eq. (1). All coefficients of models implementing this justice evaluation function can be translated on a log-earnings scale using the coefficient of the vignette dimension log earnings (β^ear^) as the denominator. Such log earnings-scaled coefficients can be used to compare results between experiments with different response instruments and response scales. Moreover, since small differences on a natural-log scale approximate rates (ln(a) – ln(b) ≈ *a*/b – 1), coefficients can be interpreted as rates or percent changes (i.e., rates * 100) if the coefficient is not too large (e.g., < 0.2 or smaller than 20%).

## Implementing the generalized Craggit model in the SOEP-Pretest 2008

In the following section, we describe how we implemented the generalized Craggit model introduced above for our analyses of the factorial survey experiment in the SOEP-Pretest 2008.

The COV in Eq. (7) enables high flexibility in the expression of the heterogeneity of rating behavior at the respondent level. As a starting point to identifying a parsimonious specification for this COV, we implemented a generalized Craggit model containing fixed effects for all vignette dimensions and indicators for the vignette decks, in addition to the respondent-level random intercepts. Our first analysis showed very strong negative covariances for the random intercepts of the Craggit selection components—ε_i_^ml^ in Eq. (3) and ε_i_^mh^ in Eq. (5)—as well as for the random intercepts of the Craggit truncated regression components—ε_i_^tl^ in Eq. (4) and ε_i_^th^ in Eq. (6). Consequently, we decided to model the random intercepts of the four Eqs. (3) to (6) using only two random effects and two additional coefficients, i.e., ε_i_^mh^ = β^ml^ * ε_i_^ml^ and ε_i_^th^ = β^tl^ * ε_i_^tl^. Thus, a more parsimonious specification of the COV in Eq. (7) consists of four random effects: one for Eq. (1), one for Eq. (2), one for Eqs. (3) and (5) and one for Eqs. (4) and (6). Out of the six covariances among these four random effects, only three were significant. Hence, we restricted the covariances that were not significant to zero, leaving us three covariances to estimate.

Building on this parsimonious specification for the COV in Eq. (7), we implemented three parameterizations for Eqs. (1) to (6) of the generalized Craggit model. This first parameterization places no constraints on the parameters in Eqs. (1) to (6). We call this parameterization “generalized Craggit model without constraints”. The second parameterization constrains all parameters in Eqs. (1) to (6), except the fixed intercepts, to be equal across equations. We call this parameterization the “constrained generalized Craggit model”. The third parameterization constrains the parameter for the vignette dimension log earnings only to be equal across Eqs. (1) and (2), Eqs. (3) and (5) as well as Eqs. (4) and (6). Furthermore, it constrains the parameter for the vignette dimension occupational status only to be equal across Eqs. (1) and (2) as well as Eqs. (3) to (6). Dropping the other constraints on these parameters optimizes the fit of the model. Thus, we call this parameterization “optimized generalized Craggit model”.^8^

Table 2 reports the model fit statistics for these three different specifications in comparison to those of a standard hierarchical linear model.^9^ The AIC and BIC of the hierarchical linear model are almost twice as large as those of the generalized Craggit model, indicating a much better fit of the latter model. Furthermore, the comparison shows that it is possible to fit a parsimonious version of our generalized Craggit model. While the optimized generalized Craggit model only has three parameters more than the constrained generalized Craggit model does, its BIC is lower than that of the generalized Craggit model without constraints which contains 115 parameters. Consequently, we used this optimized generalized Craggit model for most of our analyses of the factorial survey experiment in the SOEP-Pretest 2008. For further details on the substantive findings, see [11].

**Table 2:** Model fit of hierarchical linear and generalized Craggit models^a^.

Model	Log-likelihood	Parameter	AIC	BIC
Hierarchical linear	−133,557	27	267,169	267,390
Generalized Craggit without constraints	−73,898	115	148,026	148,968
Constraint generalized Craggit	−75,426	40	150,932	151,259
Optimized generalized Craggit	−74,069	43	148,224	148,576

a Models include fixed effects for all vignette dimensions and differences between vignette decks. *Source*: Own calculations based on SOEP-Pretest 2008 (N_vignettes_ = 26,650; N_respondents_ = 1066).

## Declaration of Competing Interest

None.

## Appendix Supplementary materials

Image, application 1

## Footnotes

1 A factorial survey is an experimental design aiming at measuring attitudes, judgments, beliefs, opinions, preferences, intentions or decisions. The experiment consists of – typical textual – scenarios (called vignettes) combining several treatments (called dimensions) with controlled varying doses (called levels) [2]. The vignettes are fictitious descriptions of situations or objects and are used as stimuli which are evaluated by the respondents. Dimensions are attributes of the vignettes. Levels are categories of the dimensions.

2 Alternatively, [16] use a multinomial logit model to analyze the earnings justice attitudes in the SOEP-Pretest 2008 data, focusing on the differentiation among “unjustly too low”, “just” and “unjustly too high”. However, such an approach neglects the additional information of the more fine-grained ratings.

3 Prior research has already used standard Craggit models to analyze factorial surveys with censored vignette rating distributions [1,4].

4 Using such a generalized instead of a standard ordered probit model enables a more flexible specification, in which coefficients can differ between the ordered categories [17].

5 This parameterization using log earnings is based on the justice evaluation function developed by Jasso [6,8]. We explain the utility of this parameterization below.

6 To stick with the rule that positive coefficients express that earnings in vignettes are “unjustly too high” the estimates related to equation (5) have to be multiplied by -1. This multiplication by -1 also has to be applied for parameter constraints applied to equation (5).

7 The covariances between respondent-level random intercepts and vignette-level error terms are zero due to the random assignment of vignette decks to respondents [16].

8 Table A1 in the supplementary materials show the COVs of these three different parameterizations. Overall, the respondent-level heterogeneity expressed by the random effects and their covariances is quite similar for all three parameterizations.

9 Specifically, we use the Akaike information criteria (AIC = −2 * log-likelihood + 2 * number of parameters) and the Bayesian information criteria (BIC = −2 * log-likelihood + ln(number of observations) * number of parameters) to compare the fit of the models. Lower AICs and BICs indicate a better model fit. In comparison to the AIC, the BIC penalizes more strongly for adding additional parameters to the model.
