# Supplementary material for: Analyzing rating distributions with heaps and censoring points using the generalized Craggit model
Source: MethodsX. 2020 Mar 19;7:100868. doi: 10.1016/j.mex.2020.100868 (PMC7155229; doi:10.1016/j.mex.2020.100868)
Supplement: Supplementary file 1 [file mmc1.pdf]

Supplementary materials

A 45-year old woman, married, with two children,
and a husband who does not have own income,
she has vocational training and
works as a hairdresser in a large company, which is threatened of bankruptcy,
Her performance on the job is below the average,

She earns **1200 Euro** gross income per month before taxes.

Your rating:

F 1:
From your point of view, is the gross income for this person just or unjust?

- □ Gross income is just (→ carry on with the next person description)
- □ Gross income is unjust (→ carry on with F 2)

F 2:
Is the gross income unjustly too high or too low?

- □ unjustly too high (→ carry on with F 3)
- □ unjustly too low (→ carry on with F 3)

F 3:
With regard to your personal feeling, which number between 1 and 100 describes most adequately the amount of injustice?

____

Figure A1. Translation of an example vignette from the SOEP-Pretest 2008
Source: Sauer, Liebig, Auspurg, Hinz, Donaubauer, and Schupp (2009)

Table A1. Variance-covariance-matrixes of the generalized Craggit models[a]

Generalized Craggit model without constraints						
Random Effects:	1)	2)	3)	4)	Eq.[b]	Coefficient
1) Probit: < 0 vs. ≥ 0	0.32***				(1)	1 (fixed)
2) Probit: ≤ 0 vs. > 0	.	0.62***			(2)	1 (fixed)
3) Craggit selection	.	-0.14**	2.61***		(3)<br>(4)	-0.95***<br>1 (fixed)
4) Craggit truncated regression	-1.10**	1.30*	.	250***	(5)<br>(6)	-1.02***<br>1 (fixed)
Constraint generalized Craggit model						
Random Effects:	1)	2)	3)	4)	Eq.[b]	Coefficient
1) Probit: < 0 vs. ≥ 0	0.20***				(1)	1 (fixed)
2) Probit: ≤ 0 vs. > 0	.	0.41***			(2)	1 (fixed)
3) Craggit selection	.	-0.11*	2.82***		(3)<br>(4)	-0.96***<br>1 (fixed)
4) Craggit truncated regression	-<br>1.76***	1.65**	.	333***	(5)<br>(6)	-1.03***<br>1 (fixed)
Optimized generalized Craggit model						
Random Effects:	1)	2)	3)	4)	Eq.[b]	Coefficient
1) Probit: < 0 vs. ≥ 0	0.32***				(1)	1 (fixed)
2) Probit: ≤ 0 vs. > 0	.	0.57***			(2)	1 (fixed)
3) Craggit selection	.	-0.13**	2.67***		(3)<br>(4)	-0.93***<br>1 (fixed)
4) Craggit truncated regression	-1.16**	1.26*	.	254***	(5)<br>(6)	-1.02***<br>1 (fixed)

[a] Models include fixed effects for all vignette dimensions and differences between vignette decks.

[b]Eq. = Equation

Source: Own calculations based on SOEP-Pretest 2008 ($N_{vignettes}$ = 26,650; $N_{respondents}$ = 1,066)

† $p < 0.10$; * $p < 0.05$; ** $p < 0.01$; *** $p < 0.001$
